# Comments on “Machine learning and SHAP value interpretation for predicting comorbidity of cardiovascular disease and cancer with dietary antioxidants”

**DOI:** 10.1016/j.redox.2025.103849

**Published:** 2025-08-30

**Authors:** Yuwen ShangGuan, Zhenhao Lin, Kunpeng Wu, Young-Je Sim, Weibo Xuan

**Affiliations:** a Department of Exercise Physiology, Kunsan National University, Gunsan, Republic of Korea; b Department of Cardiovascular Medicine, Changzhou Maternal and Child Health Care Hospital, Changzhou Medical Center, Nanjing Medical University, Changzhou, China

**Keywords:** Machine learning, SHAP, Cardiovascular disease, Diabetes, Dietary antioxidants

## Abstract

Cardiovascular disease (CVD) and cancer are leading causes of death worldwide, and the comorbid risk between these conditions has become an important area of public health research. Recently, a study published in *Redox Biology* (Volume 79, February 2025, 103470) applied machine learning models to explore the predictive value of dietary antioxidants for CVD and cancer comorbidity, making a significant contribution to this field. However, upon further examination, we found that the SHAP analysis in the paper did not reveal a significant contribution from baseline features, which raised concerns about the rationale for variable inclusion. Through independent replication analysis, we found that the SHAP results might be based on a model that omitted baseline features. A further comparison of SHAP results with and without baseline features indicated that baseline characteristics were far more important for disease prediction than dietary antioxidants. Additionally, the SHAP plots presented by the authors closely aligned with those generated from our replication of the "model without baseline features."

We carefully read the article by Qi et al. [1]. This study represents an important attempt to integrate dietary data into machine learning models for predicting chronic disease risk. Although the application of SHAP analysis provides valuable insights into model interpretability, our independent review and replication analysis found several key issues in the variable contribution analysis that warrant further discussion.

Firstly, we noticed that the authors explicitly stated in the methodology that 29 dietary antioxidant variables and 9 baseline features (such as age, body mass index, smoking, drinking, hypertension, diabetes, etc.) were included in the model. However, in the SHAP analysis plot presented in their results (Figure 5 in the original text), the top 15 variables are all dietary antioxidants, and no contribution from any baseline variables is observed. This result clearly contradicts current medical consensus, as baseline features usually play a crucial role in health risk assessment [2]. Failing to consider these variables may limit the model's predictive capability to a single factor. When using multivariable models that include baseline features for prediction, these variables are typically ranked at the top in SHAP analysis[[3], [4], [5], [6]]. Therefore, the absence of significant contributions from baseline variables in the author's SHAP analysis results raised doubts for me.

To verify this, we analyzed NHANES data from the 2007–2010 and 2017–2018 cycles, incorporating the baseline and dietary antioxidant variables used by the authors, and additionally examined the Composite Dietary Antioxidant Index (CDAI). Standard preprocessing steps were applied, including SMOTE to address class imbalance, Z-score normalization, and the exclusion of highly collinear variables. We then compared the five machine learning algorithms in the original text, followed by SHAP analysis. We adhered strictly to a full methodological replication and constructed two versions for comparison: one is the complete model, which includes all dietary antioxidants and baseline variables (Fig. 1); the other is a model with baseline features removed (Fig. 2). The results showed that the SHAP plot generated by the model without baseline variables closely matched the results presented in the original paper, with dietary antioxidants such as Vitamin A, Magnesium (Mg), Zinc (Zn), and Selenium (Se) emerging as dominant features. In the model that included baseline features, the top five variables were all baseline indicators (such as age, hypertension, smoking status, BMI, and diabetes), and their SHAP values were significantly higher than those of any dietary variables. This phenomenon suggests that the SHAP explanation presented in the original paper is likely based on a "model without baseline features," leading to an overemphasis on the predictive value of dietary antioxidants. On one hand, this undermines confidence in the rationale for model construction and the ranking of variable importance; on the other hand, if the model is used for clinical risk prediction or public health screening, it could lead to the erroneous prioritization of dietary interventions over traditional risk factors, misleading the priority of intervention strategies [7]. Therefore, we recommend that the authors revalidate the SHAP analysis, clearly distinguish between the full-variable model and the subset model explanations, and fully disclose the process of variable inclusion in the results and discussion.

**Fig. 1:**
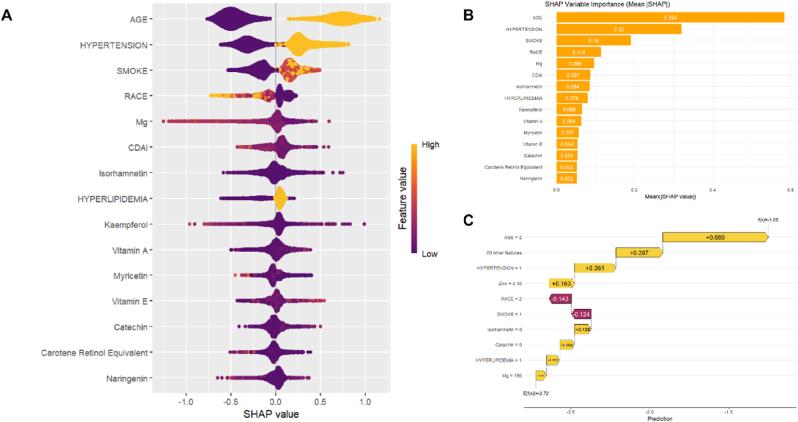
Combinatorial plots of shap analysis with baseline features. (A). SHAP summary plot. (B). Feature Importance plot. (C). SHAP force plot.

**Fig. 2:**
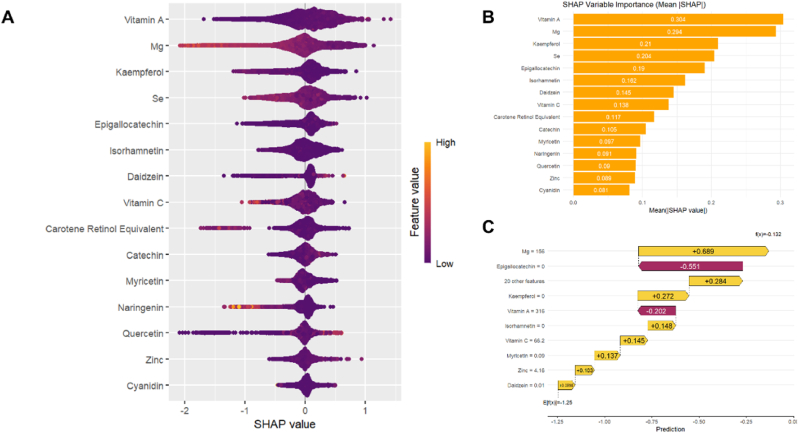
Combinatorial plots of shap analysis without baseline features. (A). SHAP summary plot. (B). Feature Importance plot. (C). SHAP force plot.

Furthermore, we also noted that the study only conducted internal 10-fold cross-validation, which can preliminarily assess model stability but lacks external validation. Considering that the NHANES data have a specific U.S. population structure and dietary cultural background, the applicability of the model to other populations remains unclear. We recommend that future research consider the following external validation approaches: Use public health survey data from different countries or regions to evaluate the model's performance across population structure differences (cross-dataset validation) [8]; NHANES data can be divided by year to construct an out-of-time validation set (time-split validation) [9].

Overall, the authors applied machine learning and SHAP interpretability methods to the study of dietary antioxidants in predicting cardiovascular disease-cancer comorbidity. This approach demonstrates innovation and provides valuable exploration for the construction of nutrition and chronic disease prediction models. We acknowledge the efforts made by the authors in data integration and model development. To further enhance the scientific and practical value of the model, we suggest that the authors explicitly include baseline features in the SHAP analysis and incorporate external validation in future research to improve the model's stability, interpretability, and generalizability.

## CRediT authorship contribution statement

**Yuwen ShangGuan:** Writing – review & editing, Writing – original draft, Methodology, Formal analysis, Data curation. **Zhenhao Lin:** Writing – review & editing, Supervision, Resources, Project administration. **Kunpeng Wu:** Writing – review & editing, Supervision, Project administration, Funding acquisition. **Young-Je Sim:** Writing – review & editing. **Weibo Xuan:** Writing – review & editing, Funding acquisition, Conceptualization.

## Declaration of competing interest

The authors declare no competing interests.

## Data Availability

Data will be made available on request.
